# Monitoring Female Fertility Through ‘Femtech’: The Need for a Whole-System Approach to Regulation

**DOI:** 10.1093/medlaw/fwac006

**Published:** 2022-04-28

**Authors:** Catriona McMillan

**Affiliations:** School of Law, University of Edinburgh, Edinburgh EH8 9YL, UK

**Keywords:** Data protection, femtech, fertility, medical devices, self-surveillance, whole-system regulation

## Abstract

Concurrent with the rise of digital health and personal health tracking technologies, a market has also emerged of products targeted specifically at women: ‘femtech’. This article is motivated by the concern that insufficient regulatory attention has been devoted to this growing market, and that extant ambiguity in the regulation of femtech leaves its users at risk of relying on technologies of as-yet unproven worth. It is posited that femtech profoundly disrupts well-established regulatory mechanisms of protection in ways that mean that these silos of protection will not be adequate. This is because regulation, as it is currently constructed, is insufficiently sensitive to feminist perspectives regarding what these technologies mean for women. As a result, the regulatory sphere in which femtech operates fundamentally fails to ensure that the health and safety of femtech users are protected as this market continues to expand. To counteract this, the argument is made that an appropriate regulatory response to femtech must respond to the distinctive unmet need in the regulation of this technological realm and the acute risk that femtech poses. This must include a multidimensional whole-system approach grounded in feminist perspectives on health, fertility, and technology.

## I. INTRODUCTION

In recent years, extensive scholarly attention has been directed towards new forms of health technology for personal use, also known as personal health tracking technologies (PHTTs). PHTTs—from ‘smart watches’ to ‘fit bits’—play an increasingly vital role in self-care and healthcare practices and have thus garnered scrutiny from those concerned with the broader implications of these technologies for issues such as self-concept^1^ and privacy.^2^ Nonetheless, insufficient attention has been drawn to the fact that many PHTTs are specifically marketed at women, known as ‘femtech’. ‘Femtech’ is a term ‘applied to a category of software, diagnostics, products, and services that use technology to focus on women’s health’.^3^ While this term was initially coined to convey a positive technological development for women, the market is now saturated with devices for personal use aimed at tracking and improving women’s biological processes, including fertility, breastfeeding, and sexual pleasure. Femtech has been discussed by scholars from fields such as sociology, anthropology, and UX design (amongst others) but it is yet to be robustly interrogated from a legal and regulatory perspective.

The focus of this article is digital technologies that are specifically targeted at processes relating to female fertility, namely menstrual cycle tracking apps, and apps that aid in contraception and/or conception. These make up a significant proportion of the femtech market,^4^ and are an acute example of the serious, life-changing effects that health technologies for personal use can have on users’ lives (eg unwanted pregnancy). This flourishing market, filled with promises of increased knowledge and control over one’s own body—often presented through pink and pastel colours and ‘feminine’ motifs (eg hearts and flowers)—sits against a darker backdrop of the broader ever-present risk of control and stigmatisation of women’s bodies within society.^5^ From the politicisation and criminalisation of women’s reproductive decisions,^6^ to using nudge to prompt ‘optimum’ decision making during pregnancy,^7^ to stigmas surrounding (in)fertility and menstruation,^8^ to inclusion in clinical trials,^9^ women today face multi-layered, institutionalised assaults on their autonomy when it comes to their health and well-being. Yet we also live in a world where women drive the majority of consumer spending, and the market for products aimed at women is booming. The femtech market is expected to be worth nearly $50 billion by 2025.^10^ Those in the field of technology, of which there are now over 200 start-ups worldwide, claim that femtech can dually assist individual women in knowing and understanding their bodies, and through the data collected enhance scientific knowledge of women’s health. The PHTT market is now replete with apps (that sometimes come paired with devices) that monitor, track, process, and advice based on data relating to women’s fertility. Choice, then, is apparently everywhere.

Set against this there are considerable regulatory concerns. For example, in past years there have been scandals concerning unwanted pregnancies after the use of a product approved by the EU as an aid to contraception.^11^ Voices have also been raised about the reliability of this technology more generally.^12^ The femtech market is hitherto operating in a dubiously regulated space. This is both a concern and an opportunity. It is a concern in as much as an absence of effective regulation is always troubling if due attention has not been given to the full range of needs, interests, and rights at stake. The diagnostic in this article is entirely to this effect. However, it is also posited that there is an opportunity in that there remain possibilities and flexibilities in designing what responsible regulation in an arena that can (and does)^13^ have benefits to human health could look like. It is to the foundations of this design that this article seeks to make a solid contribution. Underpinning both of these exercises of diagnosis and identification of potential cure is imperative to ensure that full ethical and legal interrogation takes place alongside recent examinations of PHTTs and other forms of health technology. The confluence of regulatory mishaps of the past, the fast-paced expansion of this market, growing social and ethical critiques, and the relatively light-touch regulation of these products begs the following question: What type of regulatory response does femtech require? However, to begin to answer this question we must first be aware of the nature and scope of the regulatory challenge that femtech poses. Therefore, this article seeks to address a logically prior question: What are the nature and contours of the problems we are facing, seen as a regulatory challenge?

To answer this last question, this article is divided into three main parts. First, Section II begins by providing a brief overview of femtech and engages with feminist literature to highlight why these technologies deserve urgent regulatory attention. Section III sets out the hitherto relatively uncharted legal and regulatory landscape that surrounds fertility-related femtech. This section maps out exactly what law, regulation, and policy applies to femtech in the UK. Femtech has been discussed in the realms of sociology^14^ and healthcare informatics^15^ and abroad^16^ but in the UK legal, ethical, and regulatory context. This exercise reveals an incomplete picture, both from the point of view of regulatory capture (ie having the legal instruments and mechanisms in place to capture the types of products it intends to) of fertility-related femtech in its various forms, and also concerning due recognition of the underpinning values and interests at stake. Section IV speaks to the overall conclusions that can be drawn from Sections II and III. Here, it is argued that regulation, as it is currently constructed, is insufficiently sensitive to a feminist perspective regarding what these technologies mean for women. It is claimed that the regulatory challenge at stake goes beyond one of *capture*, to one of complete *regulatory failure.* That is, there is a failure within the relevant regulatory architecture to recognise the interests, values, and risks revealed by a feminist perspective and in turn, this means that the regulatory sphere in which femtech operates itself fundamentally fails. This kind of regulatory failure speaks to the very core of what is at stake: to ensure that the interests, health, and safety of femtech users are protected as this market continues to expand. Finally, it is concluded that to provide adequate protection against varying user risks posed by this technology, a multidimensional, whole-systems approach grounded in feminist perspectives on health, fertility, and technology is urgently required.

## II. FEMTECH: A FEMINIST ISSUE?

While this article is written from a distinctly regulatory perspective, mere engagement with regulatory and legal literature is insufficient to address the research questions identified above. The entire premise of this work is that a feminist perspective and grounding is key for this analysis because technology is inseparable from morality,^17^ norms, and the social context(s) in which it operates. Femtech is unique in that it is both borne *from* these contexts and because it greatly impacts users *within* their social contexts. To explain, the invention of femtech was borne from a feminist issue, namely the technology industry’s relative ignorance of women’s health. For example, as Criado-Perez highlights when Apple introduced their health app in 2014 which claimed to be a comprehensive tracker, there was no capability to track periods until several years later.^18^ Yet, as discussed further below, not only have femtech apps and devices been critiqued as perpetuating norms surrounding women’s role in reproduction and fertility,^19^ but many of the tracking apps relied upon by users have been shown to have dubious accuracy.^20^ The latter issue came to a head in 2018 when a major Swedish hospital reported that 37 out of 668 women who sought abortion were using the Natural Cycles app as their only method of birth control. As discussed further below, not only does femtech tend to contain gendered assumptions about users’ bodies (eg menstrual cycle length, which can risk users by leading to an inaccurate prediction of the ‘fertile windows’)^21^ but it also operates within the sphere of women’s reproductive health; a sphere of healthcare blighted by criminalisation and control (in many countries) in the form of abortion laws. For this work, it is axiomatic, therefore, that femtech—as a group of technologies that are determinedly aimed at women—must be considered relative to the dynamics, norms, and varying contexts that uniquely affect women and others who use femtech.^22^ It is for these reasons that it is claimed here that femtech is undeniably a feminist issue; it follows self-evidently that the regulation of this field must also be examined through this lens. The nature and extent of this are expanded upon further below. Thus, to identify the true nature of the regulatory issues at stake, a perspective that highlights the interests and needs of the users of this technology (primarily, but not limited to women) must first be foregrounded in any discussion of the matter. It is for this reason that a feminist perspective, drawing on intersectionality, in particular,^23^ on femtech and technology is employed below.

### A. What Is Femtech?

The female body and reproductive processes within are more ‘visible’ than ever. Every stage of every process is now available to predict, track, monitor, or simply view in the abstract via medical devices (MDs), and now PHTTs. The femtech industry includes a wide range of technologies that visualise female sexual and reproductive processes, from tracking one’s menstrual cycle in a calendar app to real-time guidance for ‘kegel’ exercises. While some types of femtech go beyond the realms of the womb, the majority of it focuses on and around reproduction and fertility-related processes,^24^ despite the remit of women’s health going far beyond our ability to reproduce. Within the realms of fertility management, there are numerous apps available for download on smartphones that enable women to track every aspect of their reproductive and sexual lives including menstrual cycles, ovulation, sexual activity, contraceptive use, and any associated symptoms or side-effects of all of the latter. Some apps notify women when their period is due, remind women when they are due to take their contraceptive pill, when they are entering their fertile period, and when to change sanitary products. Some of these apps now have paired biosensing technologies, such as a thermometer, that feed data back to the app (usually via Bluetooth). Internal, or ‘insertable’ devices are available that detect changes in cervical mucus to predict fertile windows or detect progesterone levels in saliva. Femtech thus comes in the form of stand-alone apps, or wearable or insertable technology that sends data to a paired smartphone app. App users are often prompted to input personal information: from more simple facts like name and age to more complex and private matters like occasions of intimacy.^25^ The data collected via these apps have powerful potential to reveal important findings about women’s health and well-being.

It is a trope across feminist literature that women’s health has been, and remains, de-emphasised in medicine and science. For many femtech start-up-owners and developers, specifically addressing women’s health issues within the realms of PHTTs targets and therefore ostensibly mitigates this problem by emphasising the importance of women’s control over their health. Femtech specifically seeks to address some of the most time-consuming, and often difficult health processes that women have to face, namely, reproductive health. Indeed, it has been said that femtech, in a world where much of women’s bodily functions are still taboo, ‘… helps destigmatize women’s reproductive and sexual health.’^26^ And it seems that many users agree: at the time of writing, the femtech market has proliferated at an unprecedented pace.^27^ For example, menstrual cycle tracking apps are the 4th most popular among adults, and second most popular among teenage women.^28^ With so much information at the touch of a button, femtech has been hailed as empowering for women, and a vehicle for making informed choices about their health. Yet the rapid growth of PHTTs and mHealth generally, concurrently with the femtech market, has brought about a discourse that places great value on the management of one’s health via self-tracking and/or the use of monitoring devices. While positive strides have been made in the name of women’s health interests, developments in the field of women’s health must be considered against a backdrop of relegation and oppression, engrained in the history of medicine, where women’s bodies have been marginalised, underprioritised and under-researched since the practice began. For a long time, the male body was perceived in medicine as the ‘default standard’, while the biological differences that women have were often treated as an ‘… aberration or deviation from the male norm.’^29^ Many of the difficulties women continue to face in attaining healthcare derive from the structures of this history. There is also concrete evidence that these difficulties can be all the more severe and complex where women from marginalised communities (eg ethnic minorities, lower socio-economic class) seek medical attention. As the next section discusses, these power dynamics and inequitable health outcomes are undeniably intertwined.

### B. Female Health, Responsibilisation, and the Self-Surveillance Economy

Yet femtech is not merely an extension of the traditional issues and challenges that feminism faces, even in a technological context. While sex- and gender-based prejudice are of course problematic in and of themselves, the digitisation of reproductive health goes beyond the challenges with which we are already familiar. Recognising the dominance of cis-male norms that permeate the structures within society is key to exposing the problems that can be easy to overlook or ignore. Femtech has powerful potential to ‘other’ women’s bodies and perpetuates gender norms, particularly surrounding fertility and pregnancy. Women and other femtech users fall outwith the male norm that has acted as the blueprint for much of medical research,^30^ and the ‘otherness’ of bodies that bleed, gestate, give birth, and produce milk has been well documented.^31^ But that othering^32^ has led to ignorance of the challenges that women and others face where their experiences, bodies, and identities are seen as, presented as, and socially accepted as exceptional to the male purview.^33^

But how can this be when femtech promotes so many choices for women and represents a potential source of so many new forms of control for women over their bodies? Fertility-related technologies arguably operate on the assumption that users conform to particular social, sexual, and bodily norms; in other words, femtech assumes conformity to ‘the idealised subject position of the reproductive citizen’.^34^ The way in which fertility (and its importance) is socially and culturally constructed has a direct impact on the well-being of women. This is further perpetuated by femtech, as are the gender-based norms and ‘gender-based reprisals’ (prejudice, discrimination)^35^ that women often face should they struggle with fertility. Issues such as these construct ‘… female identities along artificial lines of what society and males perceive as normal.’^36^ Moreover, under the guise of promoting choice is the spectre of forces driving women to make the ‘right’ kinds of choices. Responsibilisation of women’s health when it comes to our reproductive systems is not new; indeed, it is a key factor that neoliberal political climates rely on.^37^ This responsibilisation is deepened by the fact that in the digital age, more than ever, women are encouraged to pay a high level of attention, and devote a large proportion of our time, to our fertility and reproductive systems through technology available to purchase and use from home, privately. Yet, the failure to avail oneself of new technology can easily be perceived as a failure in obligations to one’s life partner, one’s family or society more generally. Choices are certainly available, but the choice to say no might be the least available of all.

Femtech, as a means of reproductive observation and management, has created what Lupton calls a ‘fervid atmosphere of self-surveillance … in which female fertility and reproduction are experienced and performed’.^38^ This culture of surveillance-based responsibilisation derives from the private commercial sphere. After all, femtech apps placed on app stores, and products available to buy online, are all invented for *profit*. All mobile applications attached to most fertility-related femtech technologies store, analyse, and report on the user’s fertility or cycle status. Yet because of this, femtech has been critiqued as having ‘… led to novel ways of portraying and surveilling the fertile female body.’^39^ Monitoring of the female body, for fertility and reproduction, now occurs in an additional context, outside of the clinic, in-app users’ homes, places of work, and so on. It is well documented that the issues women face in achieving equality in healthcare are only made more acute when operating in the private commercial sphere (which femtech presently does), namely the so-called free market.^40^ Indeed, seen against this body of literature, femtech is a key example of the contradictory impacts that globalisation and neoliberalisation can have on women, where on the one hand they are enabled to work and participate in the economy in ways previously unavailable to them, yet, on the other hand, they are also simultaneously being dominated and devalued by the market.^41^ Simple but enduring examples of the latter range from the prevalence of unequal pay for equal work, to the booming beauty and cosmetics industry which continues to pressure women to improve their physical appearance.^42^

There are two strands of feminist perspectives on women’s decision-making within patriarchal capitalist societies that are especially illuminating, one being that women are exercising free choice and making autonomous decisions about products or procedures they use,^43^ and another that women are victims of a ‘false consciousness’^44^ and, relatedly a post-feminist media culture.^45^ It has been well documented that feminist notions are appropriated in media and advertising as a marketing tool (a pseudo-feminist veneer), which has begun to dominate how products are advertised and sold to women. They range from ‘empowering’ presidential nail polish names;^46^ to the marketisation of women’s individualisation (the idea that we have a choice over every decision); to, most relevantly to this article, the encouragement of self-surveillance. And, as Jasanoff points out, in modern society technology rules us as much as laws do, and with all technology, we hand over power.^47^ With femtech users hand over power in terms of a vast range of reproductive matters from reliance on getting pregnant, to not getting pregnant, to their personal information, to the personal identity, to their self-concept. Indeed, while the majority of femtech start-ups are led by women, these technologies are predominantly funded by men.^48^ The power here, in terms of what does and does not get funded, rests firmly in the hands of those who do not use the technology or have an experiential understanding of the reasons why one might want to use it. It is arguable that the competing perspectives of women as autonomous persons in capitalist structures, and women as dominated by capitalist structures, may co-exist. That is, it is possible for something, for example, cosmetic surgery or makeup, to be both empowering for individuals who choose to use it while also enforcing misogynistic, hegemonic ideals.^49^ As with all online information, femtech can dually act as a help and a hindrance to female health and social interests; both a system that can be accessible, supportive, and offer a sense of community, but also, as the following discussion explores, a network of false and unverified information, data gathering, and profiteering. While the choice to use a particular femtech product may be mindfully made, perhaps with full awareness of the patriarchal structures, this might simultaneously enforce and impose various oppressive biases that have historically plagued women. Feminist literature has discussed the effects of internalised oppression at length, and while there are multiple positions on the topic there appears to be consensus from relational autonomy perspectives that practices and socialisation occur which oppress women in a way that diminishes if not undermines, women’s agency.^50^ Under the facade of liberation, femtech, therefore, has the potential to ‘oppress and quantify’ women.^51^ For example, by using these technologies, users of femtech are enrolled into the data economy in which their data are collected, used, and processed, more often than not for profit. The concern here is not profit *per se*, but rather that profit motives become the drivers of why data are collected and how the data are used. Allowing a relatively free market here allows, and profits from, the promotion of an idealised female who ‘confirms to the broader idealised health-promoting citizen’.^52^ This is one of many longstanding ‘normative precepts’ surrounding women’s health, sexuality, and self-responsibility in reproduction.^53^ These precepts, it is argued here, are in some ways only exaggerated by the culture of tracking and monitoring encouraged by the purchase, download, and use of femtech.

Power dynamics of institutionalised patriarchy and other issues, mixed with profit motives, mean that issues such as these are even more deeply entrenched in a new age where we are now, more than ever, set up as citizens who are responsible for self-monitoring. If from a regulatory and legal perspective, it is law’s purpose to protect us from those structurally imbued power dynamics (which normatively, it is claimed here that it is), this leads to the question: *who* has the power here? While we hand over power to the technology itself, it is, to state the obvious, actors in any given system who control the design, dissemination, and use of any information input into femtech. In handing over that power, we hand over a degree of control over our decisions, our data, and so on. Yet, identifying the ‘gatekeepers’, as it were, in this industry, where power is dispersed amongst an unknown and unknowable range of actors is an increasingly complex and unrewarding task. For example, doctors have been described as ‘gatekeepers’ to abortion,^54^ a clear example of where female reproductive choice is tempered by patriarchal power structures. However, in the case of femtech, the dynamics of power are extended among a much wider, and as we shall see below, less regulated and less accountable range of actors. From a regulatory perspective, then, social and market power is much more difficult to attend to when this power is so dispersed and covert. Indeed, it is not unreasonable to suggest that multiple power dynamics are involved, all potentially working against the interests of women who seek to use femtech as a means to take control over their reproductive lives. A range of parties are involved in femtech, from design and development to testing, to data processing, and so on. If to properly promote women’s autonomy over reproductive choices made when using femtech, then we must enquire how those choices are influenced, limited, and/or controlled within the dynamics at play. Yet knowing who holds the power in a vast network of femtech developers, creators, and data processors is difficult in the digital age. The hidden identities of actors in that network may make their power very wide-ranging and diffuse, and this is all the more concerning.

Yet, what might the importance of autonomy, in this power dynamic, mean for regulators? To use the example of data (see Section III) one question that arises is: how far does the regulatory framework surrounding femtech allow control over the processing of user data? This is difficult to know, yet potentially important where sensitive and private data about reproductive decisions are being processed. Yet data is only one aspect of the issues described here. For example, another issue that this analysis raises is: how much control and freedom are women and users given to rely on the information and recommendations femtech makes about their health? These risks are of regulatory concern particularly where users’ physical health and safety come into play, but normatively, it is argued here that it is the job of regulation to go further to protect autonomy in its broadest sense because of the social, gendered, and psychological impact it can have. If technology does not work or work for a particular group of users, then users have no meaningful choice. This is explored in the next subsection.

### C. We Cannot Have ‘Technical Accuracy’ without Recognising Diversity

Technology is neither amoral nor apolitical, and extant prejudices and discriminations have been transferred into the design and dissemination of many technologies,^55^ including femtech.^56^ To be clear, it is not claimed here that the transference of biases into technology is necessarily intentional *per se*; inadvertent discrimination such as unconscious bias (for example) permeate social and institutional structures. Yet, like the following, the potential for the transfer of gender bias into femtech’s design may be seen in at least two stages.^57^ First, how data are gathered to create the technology (ie by basing data relied upon from a limited group of persons).^58^ Secondly, algorithms that interpret this data (and feedback into the apps) may have bias built into their design.^59^

The tracking of fertility data is not new to women. For centuries women have tracked their menstrual cycle, and more recently their body temperature to achieve contraception or (avoid) conception.^60^ Yet fertility tracking apps have led to numerous inaccurate predictions and many unwanted pregnancies. Studies have found that many popular period apps fail to predict women’s fertility accurately.^61^ For example, a review of 73 menstrual cycle tracking apps found that none could correctly predict ovulation.^62^ Reliability issues are not unsurprising given that according to one study, only 5% of femtech apps cite medical literature.^63^ While adding more regulation about data accuracy might seem like an appropriate and adequate protective mechanism to prevent this, unfortunately, the answer is not so simple because accurate femtech requires the recognition that women are not uniform. Indeed, as demonstrated below, what counts as ‘data’ about ‘women’ is an incredibly complex and heterogeneous undertaking. While the reliability of fertility apps has also been proven to be questionable, beyond that, it does not recognise the diversity of women and other potential users of femtech.

The issue of accuracy is complexified by the fact that not all users can input metrics that align with their bodies. Like any technology, the apps are only as reliable as the information that is put into them.^64^ Unless the user inputs incredibly detailed information regularly, the efficacy of the apps is likely to be considerably reduced or rendered ineffective. Relatedly, these apps do not work for all women/users of femtech. Menstrual cycles can vary greatly in length, and assumptions integrated into app design about ‘normal’ cycles can lead to poor advice and inaccurate results. The technical parameters on which many femtech apps related to fertility rely fall within a very limited range, often based on averages or a notion of the ‘norm’. For example, 28 days is often deemed the average menstrual cycle length, whereas it is recognised that cycles can range between 21 and 35 days. But the limiting ranges provided within these apps go further than limiting users in terms of usability and accuracy. By limiting processes such as menstrual cycles to a certain number of days, these apps can ‘other’ women who do not feel that their menstruation, for example, falls within the app’s prescribed ‘normal’ range.^65^ Beyond telling women what data on their reproductive biology should look like, in some cases apps make normatively loaded prompts about reproductive goals. For example, Corbin cites an app that shows a smiley face when a woman misses her period, suggesting she should be happy she might be pregnant. Some apps even suggest how much sex a woman should be having to become pregnant.^66^

The issues surrounding the perpetuation and entrenchment of norms in the sphere of reproductive health is intensified by the industry’s apparent ignorance^67^ of the fact that womanhood is not a homogenous universal experience.^68^ For example, the gender identities of those who can carry children has expanded beyond ‘woman’ or ‘female’, the health and bodily processes of each user can differ widely, and the socio-economic status of those who might use this technology is less and less limited to the middle and upper classes. ‘Women’ do not have one universal voice and set of interests, as feminist literature on intersectionality demonstrates; different factors (eg health metrics in an app) affect different persons due to race, gender identity, socio-economic status. This means that not only is femtech usable for select groups, where it is used it may give inaccurate results due to the limited range of data it relies on and feeds back to itself. While some companies have made positive strides by providing gender-neutral language,^69^ the risks presented go beyond non-inclusive language. It has been noted that the risk to users presented by inaccuracy surrounding the user data also presents broader social risk; ‘context-free data is ripe for misinterpretation’.^70^ Where data are collected from apps and passed on to third parties, in some research cases, the results which feedback directly into the market tend to be based on a limited part of the population thus ignoring those who cannot use the apps (but may want to) because of their limited scope in terms of, for example (but not limited to): recognising length of mensuration, those who do not menstruate, those from different racial and cultural backgrounds, and the experiences of trans and non-binary users. Indeed, there is evidence that while data on the fertility of cis-women are readily available in various contexts, there is a dearth of this kind of data for trans and non-binary people.^71^ There is also evidence to suggest that due to the homogenous nature of digital technology development, there is also a great risk of exclusion and bias being built in for those with intersectional characteristics, for example, race and class.^72^

There is a myriad of issues with the assumptions made in the design of these apps

… femtech developers have created products that categorise women and limit their data input to a range of normal values, even if the woman’s unique, personal biology falls outside the artificial normal range.^73^

These technologies seemingly operate on the assumption that users conform to particular social, sexual, and bodily norms; this creates two issues (i) accessibility for those who fall outwith those norms, and (ii) as above, a self-perpetuating re-enforcement of norms because the data the apps collect (only enabled for those with bodies they acknowledge in their app) are collected and used for future developments. These apps are generally based on general assumptions about women’s bodies, with very little input on the wide variety of ways in which female processes can occur (eg length of the menstrual cycle). It is clear that apps fail to account for women’s design needs (eg interface, usability for a range of users), and differences in bodies and hormones. Further, the above concerns regarding what counts as ‘data accuracy’ evidences femtech industry’s clear lack of understanding of, and responsiveness to, its diverse (potential) user base, some, but not all, of which identify as women. The feminist issue here is thus clearly one that goes beyond queries about simple technical accuracy for a limited group of users that developers have determined as ‘the norm’. Rather, to achieve the true accuracy and reliability that femtech users need, the evidence base needs to be gathered from women and users in all of their diversity. A further key issue for any regulatory response then, is *who* uses femtech? In order words, who are the ‘fem’ in femtech?

### D. Who Are the ‘Fem’ in Femtech?^74^

As Butler highlights, ‘women’ is a category more complex than the singular identity often afforded in politics and language.^75^ Femtech, distinctly aimed at ‘females’ arguably assumes this singular identity. While the literature on femtech, and the technology itself, oftentimes only refers to ‘women’ as people who identify as such are the majority user of femtech (a shorthand for female tech), from an intersectional perspective it is important also to acknowledge different interests may be in play for users that do not identify as women. Women and other users of femtech are made up of a diverse group of different cultures, races, gender identity, and socio-economic classes. Each of these factors has been shown to have a profound effect on health outcomes, yet as demonstrated above the evidence base for much of femtech is limited to a ‘norm’, whatever that may be. Technology affects, shapes, and even controls different groups in society in different ways; it is, therefore, axiomatic that women (or a user of femtech) who come from a variety of backgrounds are also affected differently. This is not to say that there are not broad concerns that affect all users (eg a lack of reliability), but the *degree* of effect on subsets of femtech users undoubtedly varies in nature and extent.

Yet, from a regulatory perspective, if the range of consumers likely to use femtech is far broader and diverse than has been first imagined, then this has profound implications for the technologies in question, their efficacy, their value, and their impact on the lives of users. All of this, in turn, raises crucial regulatory challenges if we are committed to regulating femtech in ways that genuinely protect and promote all users. But what does this mean beyond requiring a stronger evidence base? This question is important because, as highlighted above, the threat to women and other users is more than one of reliability. This analysis has implications for several areas of regulation, and the problems cannot be ‘solved’ by one regulator alone. The regulatory environment, then, must cast a net more widely than we might first imagine. Indeed, it might require multiple regulators or regulatory actors to be involved across a range of sectors, and not merely within one sphere of influence such as healthcare or software development.

### E. Interim Conclusion

Despite the positive attributes and popularity of femtech, which is useful, convenient, and even liberating for many of its users, the digitisation of fertility also comes with great costs to the rights and interests of women. As described here, in many ways femtech devices and apps are replicating and entrenching gender norms and social inequalities through heightening the responsibilisation of women’s health by entrenching technological self-surveillance as a norm. Further, there is the issue of lack of equality of access and suitability for users that do not fall within the bodily norms that fertility apps prescribe. The assumptions, design features, and data on which some femtech is based, as Corbin points out ‘… can be perceived as failing women and acknowledging the full scope of women’s needs.’^76^ However, beyond all of this and as argued in this section, no picture of the interests at stake with femtech is complete without accounting for the multi-layered and wide-reaching structures that affect ‘women’ (in the most diverse sense). For answers, we need to look beyond traditional spheres of enquiry. If systems of power pose a threat to femtech users’ interests and health—and if those power imbalances are diffuse and wide-ranging across multiple sectors—then any regulatory response needs to look at the entire potential regulatory ecosystem (not just the health regulation sphere) that surrounds Femtech. As discussed further below, this requires, for example, also looking towards marketing and advertising.

So, is regulation responding, or able to respond to the points for regulation raised above? To what extent does our existing regulatory framework capture or reflect these concerns and points of regulatory attention? To answer these questions, the next section, therefore, scopes the nature and extent of the regulatory attention given to femtech thus far in the UK as a typical exemplar of the general international approach.

## III. FEMTECH: A REGULATORY ISSUE?

Due to the regulatory, social, cultural, and institutional structures in which femtech operates, these products pose clear fields of physical and socio-cultural risk to users. To be clear, as evidenced by the discussion so far, the effect on health and well-being in this context is 2-fold. First, as described above there is potential to have effects on the female and other femtech user populations more holistically; gender norms have been shown to have an impact on health and well-being.^77^ Secondly, and perhaps more tangibly, further there have been demonstrable impacts on individual users in the form of inaccurate advice and results, which can lead to traumatic experiences such as unplanned pregnancy.

As this section explains with femtech we simultaneously face both (i) existing regulatory challenges associated with the regulation of health in the broadest sense and (ii) a new set of regulatory challenges (given the analysis in the previous section). On the former, in some ways, femtech is a reflection of the traditional challenges we face as an amalgam of existing issues within the regulation of health and medicine more generally, and for femtech more specifically there is a lack of connectedness in the regulation of human health;^78^ institutionalised male-dominant perspectives in health, and slow responses to new developments in technologies. As to the new regulatory challenges, these occur because femtech is a unique form of PHTT where particularly acute consequences and outcomes can occur, from exclusion and oppression to more tangible problems such as unwanted pregnancy. It is claimed here that it is important to shed light on the entire system, or regulatory ecosystem, in which femtech operates to truly understand the nature and extent of the regulatory problem. So how has the regulatory system responded to this challenge, if at all? The following highlights the few, but sporadic ways in which fertility-related femtech is captured by the existing regulatory system. It finds that the risks outlined in Section II have been left unmitigated by the multiple regulatory frameworks that surround femtech. Yet capturing certain risks posed by femtech, for this analysis, is only part of the picture. For this work, we need to look beyond atomistic, siloed approaches to regulation, because systematic oppression requires a systematic response. The first of these silos relevant to femtech is data protection.

### A. Protection of User Data

The data collected and processed by femtech apps have powerful implications for the privacy interests of its users. This subsection discusses the application of the General Data Protection Regulation (GDPR) in the UK^79^ (tailored by the Data Protection Act 2018) and the guidelines set by the Information Commissioner’s Office (ICO). The GDPR protects and regulates the processing of personal data, a subset of which is ‘special category data’^80^ (which includes data concerning health, sex-life, and sexual orientation) that captures much of the kinds of data involved when women use femtech apps. However, there is no specific express category relating to the full gamut of fertility data. Fertility-related data are not mentioned explicitly in the ICO guidelines or in the UK legislation implementing the GDPR. One might reasonably assume that it falls under ‘sex life’ and ‘health’ data, yet while health data are defined as ‘data concerning health’, meaning personal data related to the physical or mental health of a natural person, including the provision of healthcare services, which reveal information about his or her health status,^81^ ‘data concerning’ sex life are not defined. As such, there are no specific regulations for fertility data when they are collected and processed by those other than health and medical clinics.^82^ While superficially this might seem unproblematic, it is a concern for this enquiry because, as the following explains, there is a multi-layered complexity to fertility-related data which arguably needs to be reflected in regulation that affords its protection.

While data collected by femtech apps may naturally be viewed as ‘health’ data, and thus sensitive, the nature of this data is arguably more complex and falls into multiple categories,^83^ not all of which have ‘special’ protection:

[T]he data collected by fertility apps (and other technologies) is a complex combination of health, medical, sex life and lifestyle – which may reveal eg religious and political views or sexual orientation. For example, sexual activity during menstruation is prohibited in some religions and cultures. Such patterns would easily show in fertility records.^84^

Data collected and processed by fertility-related femtech products are a blur of categories. Placing them under the heading of ‘health’ without any context therefore does not reflect the nuance and delicacy required for what is for most users, such important data. These issues are not restricted to the femtech realm, however. Critiques regarding notice and consent, blurring of regulatory categories, and the narrow scope of data protection regulation when it comes to individual and societal harms have also been recognised in the realm of digital health more generally.^85^ Yet, there is a very particular and potentially severe risk here. Data protection is often conceived of as a ‘catch-all’, that applies to everyone because it is concerned with identifiability and controlling the processing of data. However, to return to the intersectional analysis discussed earlier, what data protection is arguably lacking is the different and disproportionate ways in which different types of data can disproportionately affect different groups. For example, data about a particular social group could be highly stigmatising compared to genetic data from another group. This not only poses a risk to privacy but also the autonomy of users, to some of whom the sharing and processing of their data may be more important than others. These nuanced but crucial differences for those who share their data are not presently reflected in data protection law. To explain, a clear instantiation of this is the lack of adequate protection in the law relating to very sensitive data such as ovulation and sexual activity. When uploading a new app to the app store, it is very easy to categorise apps in a way that (either knowingly or unwittingly) avoids the scrutiny attached to ‘health and medical’ apps. This miscategorisation allows for potentially sensitive data to ‘slip through the net’ by being collected in an app categorised as one that tends to come with less stringent data processing rules (relative to health/sensitive data) such as ‘games’.^86^ Evidence suggests that app miscategorising regularly occurs within app stores on smartphones.^87^ The data collected by these apps are often circulated for commercial gain and are used and reused by a range of actors and agencies for a range of purposes.^88^

A recent empirical study of privacy risks associated with 30 top ‘fertility apps’ by Mehrnezhad and Almeida found that many apps do not comply with the GDPR and ICO guidelines.^89^ They found that 12/30 apps did not present any privacy-related information at all when opened for the first time. Several breached ICO guidelines by not including more than one option for consent to privacy terms (ie Agree *and* Disagree), and 18 out of the 30 apps had a ‘take it or leave it’ approach which meant that users had to accept one set of privacy terms or not use the app at all. They also found that several apps fail to meet the requirements of the GDPR by continuing to process personal information, or track user data,^90^ in ways in which users may not be aware:

Women may be aware of and have consented to some of these uses as agreed on or requested when first using the chosen app, but have no knowledge of others (such as some sensors on their devices and tracking data).^91^

This, they conclude, shows that standard practices in fertility apps can ‘easily violate the law’.^92^ This easily puts users at risk of breaches of privacy. While this breaches guidelines, if not data protection law, these violations also mean that many users may sign up for something that does not provide enough information on the processing of their data. This is a key problem with the power dynamics at play here, whereby women and femtech users often feel that they *need* some sort of way to track fertility-related processes in their bodies and are left with no choice but to click ‘accept’.^93^

Data protection is a mechanism that considers how individuals are confronted with power imbalances when their data are processed. Yet once collected, it is very difficult for individuals to control their data. For example, while data protection regimes mandate that personal data must be processed fairly and lawfully, they do not mandate that this must always be done with the consent of the data subject. Indeed, informed consent is merely one of a long list of lawful justifications for processing data. Thus, while privacy might be adequately protected through data protection, it is not equipped to address wider and deeper concerns about control and autonomy for data subjects. Even when consent is in play, there are concerns. In terms of femtech, the ‘take it or leave it approach’ of most apps mean that women agree to the processing of their data out of a more tangible interest in monitoring their fertility. Data processing, therefore, creates risks for individuals, which data protection regulation aims to mitigate, but these risks are more acute where individuals belong to a marginalised group. Processing may therefore easily exacerbate the power imbalances within our societies.

Data protection and feminism operate with the aims of both theorising about and changing power relations and structural inequalities. While data protection regulation does recognise some protected characteristics, as this subsection explains it is arguable protection does not go far enough for femtech users; not only does femtech pose a risk to privacy, but also user autonomy and control. The regulations described above arguably do not recognise that certain types of data mean holding a particularly powerful ‘card’ against a particular group or subset of a group means that regulation cannot be a one-size-fits-all model. While the data protection law discussed here recognises that certain data need ‘extra’ layers of protection, it does not recognise that those in marginalised groups may be more impacted. The data collected by femtech have the potential to be spread far and wide (particularly if they are entered into a miscategorised app or are a complex mix of information that does not necessarily come under ‘health’), and a vast range of parties might end up processing the data. This not only has wider privacy implications but once again for individual autonomy regarding what is done with sensitive data. And from a relational perspective, it is clear that whoever is holding the data in a particular dynamic holds the power; not only power over processing but what that data are used for. This can have significant implications for femtech users where that data are fed back into the development of femtech, and femtech updates, the fact that it is often collected from a limited set of metrics (discussed in Section IIC) creates a self-perpetuating feedback loop of limited if not inaccurate evidence.

The issues highlighted in Section II, the power of developers in holding data, the limited nature of that data in terms of recognising user diversity, and the lack of provision for user autonomy when sensitive reproductive data are being processed, are hardly reflected in data protection law. In the age of the smartphone, for many, this is now one of the only ways of tracking and getting information about their bodies. The power dynamics that could be generated or disrupted by allowing flows of data about users’ reproductive activities can have a disproportionate effect on women and users in ways that are not recognised by data protection. While loopholes such as app categorisation have direct implications for the processing of data, as highlighted here, categorisation of technology and/or software that relates to health is an issue that falls under different parts of the regulatory system that surrounds femtech; one such part is MD regulation. This is discussed in the following subsection.

### B. Femtech as a Medical Device?

MDs, as regulated by the Medicines and Healthcare products Regulatory Agency (MHRA), are defined as any ‘instrument, apparatus, appliance, software, material or other article … used alone or in combination, together with any accessories’ for diagnostic or therapeutic purposes.^94^ The term ‘medical device’ originally connoted devices used as part of diagnosis and treatment by doctors (eg an intrauterine device), rather than to software^95^ and devices for personal/private use like femtech, but as the medical technology market has expanded so has the remit of the MHRA. The MHRA has guidance available which includes reference to software made with the intention of controlling conception which ‘is applicable to standalone software and apps placed on the Great Britain [“GB”] market’.^96^ MD regulation in the UK derives from EU law,^97^ but any changes from hereon to MD regulation in GB will be brought through the powers granted in the Medicines and Medical Devices Act 2021, which was created to supplement the 2002 regulations and provide a framework for MD and medicine regulation post-Brexit. This subsection focuses on GB, as there is a different regulatory system for medical devices in Northern Ireland.^98^ In brief, according to guidance available from the MHRA, if a product is *intended* by the *manufacturer* for the purposes of *controlling conception*, it should be classed as a IIb MD.^99^ As the following discusses, there are three notably ambiguous terms,^100^ which are all required for femtech to be captured by regulation. Each of these are overtly tied to maintaining the power imbalances described above, whereby the users may still be put at risk, particularly when it comes to reliability and accuracy, should a femtech product evade MD regulation.

First, there is also significant ambiguity surrounding the meaning of ‘manufacturer’, the person(s) whose intention matters for the purposes of the regulations.^101^ Yet tracing a sole manufacturer or company in the context of fertility-related femtech is less straightforward than traditional physical products, because most (if not all) of fertility-related femtech are software-based. As Downey and Quigley point out, not only is setting up apps easy, which means that anyone can create them, but their development tends to be designed, updated, and monitored on a global scale. This means that ‘[t]he collaborative nature of many projects may mean there is no readily identifiable natural person to whom responsibility could be (easily) assigned.’^102^ This means that the power dynamic is dispersed amongst a broad range of persons with varying accountability. Moreover, uncertainty surrounding the meaning of terms such as ‘intended’ and ‘manufacturer’ allows for apps, in particular, to fall through the regulatory net depending on how they are categorised and marketed. For example, as discussed above, there is empirical evidence that miscategorisation of femtech regularly occurs in app stores. Secondly, ‘intended’, according to the guidance, is determined by the device’s instructions, labelling, and any promotional materials.^103^ There is little clarification beyond this. This shows little attention to ways in which companies can get around the regulatory requirements that are present with ease (with or without intention), despite having potentially devastating consequences. The intention has little importance where the product may still be used differently. Finally, ‘controlling conception’ is defined in the MHRA guidance as including ‘devices that claim to be directly able to make pregnancies more likely or to be able to prevent pregnancy’.^104^ It goes on to explain that:

Examples that may be devices include:

- Apps intended to facilitate conception and enable contraception based on basal body temperature.
- Stand-alone software application for conception and contraception purposes using data entered by the patient.

Examples that are unlikely to be devices include:

- Apps and software that simply replace a written diary/log to track or display data related to a woman's menstrual cycle.
- Apps and software that just provide tips or advice^105^

The guidance’s framing of ‘may’ be an MD is unclear; there is little in the way of clarification beyond what is written there. The explicit inclusion of ‘control of conception’ within the regulations and the MHRA’s remit is a good start, yet therein lies a distinct irony. As highlighted above, femtech has the potential to limit, if not strip, women of reproductive autonomy by not protecting user interests and needs. Ambiguity, in this context, thus not only poses a risk of not capturing much of femtech, but that risk is dangerous because in practice the lines between what can and cannot control conception are rather blurred. If anything, there is a spectrum of apps that relate to fertility and the control of conception. As described above, these range from providing information on menstrual cycles on one end to tracking menstrual cycles in the same way one might in a calendar, to providing analysis on that tracked data, to doing the latter with the aid of a device such as a thermometer. It, therefore, is not clear whether technologies that may be used as a contraceptive or conceptive method (but are not marketed or ‘intended’ as such) would still fall under the MHRA’s remit. This guidance only seeks to address one very particular kind of femtech (that which clearly *intends* to control conception) yet there are other ways in which these apps and devices can pose risk. For example, some apps for menstrual cycle tracking offer information about the user’s ‘fertile window’, which leaves users open to reasonably to assume that unprotected sex outside of that window will not result in pregnancy. The danger that this poses is evidenced by the fact that some apps, for example, Clue,^106^ recently updated their software to warn users that their avoiding ‘fertile window’ is not enough to prevent conception.

We need more certainty as to whether these apps are *definitely* classed as MDs by the MHRA. But the issue herein is not just one of uncertainty; for femtech (amongst other devices) there are issues beyond whether a product is captured by the regulation. It is of note that even where products are captured by MD regulation, it is yet to require that products show more than safety and *performance* (rather than ‘efficacy’),^107^ which has been controversial because semantically it means a device does not have to perform well. Further, the framework’s reliance on a declaration of *intention* by a potentially globally spread and unknowable group of manufacturers displaces the accountability for the creation of tech that women can (and evidenced by Clue’s update, clearly do) use for the control of conception. It firmly strips femtech users of autonomous decision-making about whether or not to have children, and firmly leaves the power and choice squarely in the hands of femtech whether or not to decide to include warnings regarding loopholes such as the one described here. It is clear that in allowing loopholes such as these, the very design of the MD regulatory regime is to encourage *more* entry into the market.

More detailed guidelines that capture products more fully are required at a minimum, but a larger issue that needs to be addressed is that it is also unclear whether classing fertility-related apps as MDs in general terms is helpful when there are user-specific needs that also require attention. The burden of protecting user interests and needs cannot wholly rest on MD regulation. There are multiple other issues with femtech, as highlighted in Section II, that go beyond their remit of user safety. Yet aside from data protection, there has only been one other regulatory sphere involved with femtech, and interestingly it goes beyond the realm of the health regulators: advertising standards.

### C. Beyond the ‘Traditional Regulators’: Advertising

These regulatory spheres of MD and data regulation, viewed in isolation above, are only part of a broader regulatory picture. We cannot and should not be atomistic in our approach to the regulation of femtech. Regulators tend to focus on ‘silos’ of health-related objects (eg spheres of regulation like devices, tissue and data)^108^ but femtech is a particularly clear instantiation of the new issues that modern health, which is so increasingly intertwined with personal technology, which is inherently privately bought creates. While the private commercial sphere has always been a concern for authors on medicine health, what is particularly concerning here is that a new form of healthcare with such multifaceted layers of risks has jettisoned users into the so-called free market, which has always been incredibly guilty of transgressing women and other vulnerable groups’ interests and rights. If the regulatory landscape that surrounds femtech is wider than one might think, just how far does this regulatory ecosystem extend?

The MHRA’s recent guidelines might seem a sensible step because it captures apps that claim to be contraceptives or facilitators of conception, and it is also the only regulator that addresses femtech from a medical and well-being standpoint. While data protection law, in all its forms, has always governed the processing of data in the UK, until more detailed recognition of apps for contraceptive control was detailed in MHRA guidance,^109^ the only recourse or protection for users of femtech would have been the Advertising Standards Authority (ASA). The serious consequences of these apps, operating in a relatively free and unregulated market, appear to be an afterthought after the scandals that occurred with Natural Cycles,^110^ and other apps.^111^ For example, before the 2018 scandal, in 2017 an advertisement by Natural Cycles was taken out of circulation in the UK after complaints (which in the end were upheld)^112^ were made about the veracity of the claims in the ad, which was taken down shortly after. Interestingly, at this time Natural Cycles was certified as a IIb MD by the EU. Not only does this form of regulation act *after the event*, but it requires users to be knowledgeable of the information claimed by an advertisement, and for users to have the time and wherewithal to complain in order for the ASA to be made aware. And it is further clear that the regulatory hurdles required passing *before the event*, as supplied in the regulation of IIb devices, do not provide the degree of evidence or protection that users need in a technology where so much power over one’s body (ie the possibility of (not) getting pregnant) lies in an app’s analytical prowess. For the future of advertising requirements, it is possible, given the analysis offered here, that not only should advertisements require accuracy and flagging of an evidence base, but the authority may need a higher threshold where a diverse user range may be differently affected.

### D. A Fragmented Regulatory Approach

Femtech needs more regulatory attention as a technology uniquely impactful on its users’ health. While some have argued consumer demand (ie demanding better products) is a sufficient and necessary response to some of the issues highlighted here,^113^ such a response is insufficient. The issues above are not something that can just be left to the relatively untampered PHTT market. As in any product market, responsibilising the consumer is highly problematic and potentially unethical; creating unequal situations such as this ignore the extent, complexity, and might of the power structures that uphold any given issue. In its ambiguity of application to the full range of femtech, the lay of the current legal and regulatory landscape leaves little capacity to properly respond to, or attribute accountability for the critiques highlighted in the previous section. Therefore, the risks and issues that require regulatory attention highlighted in the previous section cannot be mitigated by MDs or data regulation alone. Even if the MHRA, for example, captured fertility-related femtech in its entirety the issues of free market, profiteering, exploitation, etcetera would remain unaddressed. Yet nor can we expect the MHRA, or any of the health regulators, to be fully responsible for that. Therefore, even if this question of sufficient regulatory capture by data protection, MD, or advertising regulations can be addressed by clarifying whether femtech apps are indeed MDs, this does not fully address the regulatory problem that arises from this technology. This is because of the fundamental core values and interests that are engaged by this kind of technology determinedly directed at women. The full ramifications of this are addressed in Section IV.

## IV. TOWARDS A WHOLE-SYSTEM APPROACH TO THE REGULATION OF FEMTECH

Users must be protected where private industry and health meet because in capitalism profit always comes before rights and interests. Femtech is a complicated and nuanced technology that needs key understandings about women and user experiences, health, and interests to be reflected in products to protect them. Until we recognise and embrace the vagaries of systemic regulatory failure and consider the whole system in which these failures are occluding, we cannot ground effective and responsive future regulation in the core points highlighted above. Yet doing so is urgent, or fertility-related femtech will continue to have the power to perpetuate damaging stereotypes, harms, and discrimination against women.

To the extent that femtech, as a novel field of technology, is regulated at all, this currently happens in a piecemeal and ‘unintentional’ manner. ‘Unintentional’, here, refers to the complete lack of regulatory awareness of, and reticence to pay attention to, the fundamental rights and interests that are at stake for women and other users of femtech a class of consumer, and as a social group that has been repeatedly oppressed by social and legal regimes. The above discussion evidences that the current regulatory sphere ignores the fundamental attention and care that menstruating, conceiving, and carrying children require. Femtech thus raises important questions regarding regulatory capture, ie whether certain apps fall within the MDs regime or whether certain types of personal data should be treated as ‘sensitive’. Arguably, this problem is more acute than a lack of ‘capture’, it goes further than this; a systemic *regulatory failure* at large across the entire femtech industry. This largely takes the form of a failure to understand the nature and extent of the threats that exist as yet further assaults on women and other users of femtech’s bodies, autonomies, and identities. So how, then, do we address these multifaceted issues of oppression, gender, the so-called free market, and health?

We need to address the *whole regulatory ecosystem* that surrounds femtech because the lay of the current regulatory landscape leaves little capacity to properly respond to, or attribute accountability for the critiques highlighted here. By looking at the ecosystem involved at present, as has been done here, we understand the true nature of the problem, and only by addressing the system in its entirety can the risks and issues above be properly mitigated. Even if the current regulatory regimes become crystal clear on their ability to capture apps, for example through MDs, this does not address a deeper problem regarding regulation as a failure as a system—it does not reflect what feminist perspectives would expect and require. And unless we understand what that picture is composed of, we cannot possibly hope to begin to develop an adequate regulatory response. An understanding of the entire regulatory system in which femtech operates, as analysed here, is key to utilising that system to its fullest potential by embedding key stakeholder values and perspectives that are hitherto unreflected. This requires interconnectivity, not just in the development of these apps, and the regulation of them as part of a health regime, but also the marketing and profiteering that *drive them.* Further, the other regulatory regimes that protect us from other types of risk have nothing to do with health regulators directly (eg the ASA) but nonetheless are an important part of picturing the true nature of the regulatory challenge we face with femtech. Indeed, the full extent of the regulatory ecosystem implicated by femtech is elusive, and there may be more than the three regulators discussed here.

What a whole-system approach entails for femtech requires further enquiry, but from the above, a five-point plan (in the form of questions) for the next steps towards a whole-system approach may be identified:

1. What does a whole-system approach to regulation involve, and what does it look like?
2. How far does the system extend, and *who* and *what* are implicated?
3. Is there a hierarchy of authority within this system, and should there be? How do we get regulators to work together?
4. What are the full range of values and interests at stake, and can they be articulated and taken up by regulators (eg a concordat)?
5. What lessons can be learned from other sectors where this has been a success?

The need for a ‘whole-system’ approach to regulation in health has been recognised in the context of health research regulation,^114^ which, as part of the same broader ‘health’ ecosystem also operates within similar silos. These issues are complex and require careful and nuanced navigation in the development and dissemination of these apps.

## V. CONCLUSION

This article contributes an original problematisation of the regulatory challenge presented by femtech. It has offered a feminist analysis of the regulatory system that surrounds femtech in the UK (which is a typical example of other regimes) that goes beyond traditional, atomistic analyses of regulatory challenges. By providing a clear understanding of the challenge posed to our regulatory system, the findings here have the potential to provide a solid foundation for reform in multiple areas of law that reflects the needs, interests, and health of femtech’s users.

As described here, multifaceted problems arise out of femtech that axiomatically require a multifaceted approach, but so far the approach to regulating femtech that targets fertility-related issues has only been met thus far with the above legislation. Until this is the case, it is unlikely that femtech apps will become as effective and reliable as users need them to be. Yet the systemic problems, as identified in this article, are easier to identify than to remedy. Mitigating the risk and damage caused by historical and institutionalised bias, oppression, and the force of the free market (to name a few) would be no mean feat. Equally, in any attempt to tackle this, it would be imprudent to attempt to do so in one sweeping measure. It is highly unrealistic to imagine that a solution could be delivered under the remit of one regulator or one piece of legislation. Indeed, it would be naive to think that bespoke regulation is possible when it comes to such a complex problem.

## FUNDING

This research was funded by the British Academy, with grant number [PF20/100019].

*Conflict of interest statement*. None declared.

## Footnotes

1 D Lupton, ‘Quantifying the Body: Monitoring and Measuring Health in the Age of mHealth Technologies' (2013) 23 Crit Public Health 393.

2 See C Rosas, ‘The Future Is Femtech: Privacy and Data Security Issues Surrounding Femtech Applications’ (2019) 15 Hastings Bus L J 319.

3 ibid.

4 FemTech Analytics, ‘FemTech Industry 2021 / Q2 Landscape Overview’ (FemTech Analytics, 2021) 18 <https://analytics.dkv.global/FemTech/FemTech-Industry-2021-Report.pdf> accessed 14 December 2021.

5 See L Gurrieri, J Previte and J Brace-Govan, ‘Women’s Bodies as Sites of Control: Inadvertent Stigma and Exclusion in Social Marketing’ (2013) 33 J Macromarketing 128.

6 See the Abortion Act 1967.

7 See WHO, Global Alcohol Action Plan 2022-2030 to Strengthen Implementation of the Global Strategy to Reduce the Harmful Use of Alcohol’ (WHO, June 2021).

8 M Shildrick, *Leaky bodies and Boundaries: Feminism, Postmodernism and (Bio) Ethics* (Routledge 2015).

9 I Malhamé, R D'Souza and MP Cheng, ‘The Moral Imperative to Include Pregnant Women in Clinical Trials of Interventions for COVID-19’ (2020) 173 Ann Intern Med 836.

10 M McHugh, ‘Does Femtech Give Users Control of Their Health or Take It Away?’ (*Ringer*, 18 March 2019) <https://www.theringer.com/tech/2019/3/18/18267094/femtech-female-health-apps-menstruation-fertility-trackers-clue-glow-ava> accessed 20 August 2021.

11 L Shemtob and R Littlewood, ‘Case in Point: Natural Cycles Facebook Advertisement Withdrawn’ (2018) 44 BMJ Sex Reprod Health 316.

12 See Section IIC.

13 B Stiglbauer, S Weber and B Batinic, ‘Does Your Health Really Benefit from Using a Self-Tracking Device? Evidence from a Longitudinal Randomized Control Trial’ (2019) 94 Comput Hum Behav 131.

14 See D Lupton, *Digital Health: Critical and Cross-Disciplinary Perspectives* (Routledge 2017).

15 See M Mehrnezhad and T Almeida, ‘Caring for Intimate Data in Fertility Technologies’ (2021) 409 Proc 2021 CHI Conf Hum Fact Comput Syst 1.

16 See B Corbin, 'Digital Micro-Aggressions and Discrimination: Femtech and the “Othering” of Women' (2019) 44 Nova Law Rev 337.

17 S Jasanoff, *The Ethics of Invention: Technology and the Human Future* (WW Norton & Company 2016).

18 C Criado-Perez, *Invisible Women: Exposing Data Bias in a World Designed for Men* (Vintage 2019) 176.

19 See Corbin (n 16).

20 See Mehrnezhad and Almeida (n 15).

21 M Duane and others, ‘The Performance of Fertility Awareness-Based Method Apps Marketed to Avoid Pregnancy’ (2016) 29 J Am Board Fam Med 508, 511.

22 Notably not all users of femtech identify as women (eg trans and non-binary users), see Section IID.

23 K Crenshaw, 'Mapping the Margins: Identity Politics, Intersectionality, and Violence against Women' (1991) 43 Stanford Law Rev 1241.

24 FemTech Analytics (n 4).

25 See D Lupton, ‘Quantified Sex: A Critical Analysis of Sexual and Reproductive Self-Tracking Using Apps’ (2015) 17 Cult Health Sex 440.

26 M Simeone, ‘Can Femtech Deliver Radically Personalized Care to Women?’ (*Dataconomy*, 30 January 2020) <http://www.dataconomy.com/2020/01/can-femtech- deliver-radically-personalized-care-to-women> accessed 20 August 2021.

27 D Lu, ‘The Femtech Gold Rush’ (2019) 242 New Sci 20.

28 ‘Does DigitalHealth Technology Know Women?’ (*Medical Futurist*, 21 February 2019) <http://www.medicalfuturist.com/femtech-womens-health/> accessed 20 August 2021.

29 Corbin (n 16) 342.

30 See N Tuana, ‘The Speculum of Ignorance: The Women's Health Movement and Epistemologies of Ignorance’ (2006) 21 Hypatia 1.

31 Shildrick (n 8).

32 See S De Beauvoir, *The Second Sex* (Knopf 2010).

33 Tuana (n 30).

34 D Lupton, ‘“Mastering Your Fertility” The Digitised Reproductive Citizen’ in A McCosker, S Vivienne and A Johns (eds), *Negotiating Digital Citizenship: Control, Contest and Culture* (Rowman & Littlefield 2016) 81, 87.

35 Mehrnezhad and Almeida (n 15) 2.

36 Corbin (n 16) 355–6.

37 Lupton ‘Mastering Your Fertility’ (n 34) 88.

38 ibid 83.

39 ibid 81.

40 See R Baru and M Mohan, ‘Globalisation and Neoliberalism as Structural Drivers of Health Inequities’ (2018) 16 Health Res Policy Syst 1; S Nair, P Kirbat and S Sexton, ‘Population Politics and Women’s Health in a Free Market Economy' (2005) 48 Development 43.

41 I Chatterjee, ‘Feminism, the False Consciousness of Neoliberal Capitalism? Informalization, Fundamentalism, and Women in an Indian City’ (2012) 19 Gend Place Cult 790.

42 K Windels and others, ‘Selling Feminism: How Female Empowerment Campaigns Employ Postfeminist Discourses’ (2020) 49 J Advert 18.

43 See K Davis, *Reshaping the Female Body: The Dilemma of Cosmetic Surgery* (Routledge 2013).

44 See Chatterjee (n 41).

45 See A McRobbie, ‘Post‐Feminism and Popular Culture’ (2004) 4 Fem Media Stud 255.

46 S Hallett, ‘Kerry Washington Released a Feminist Nail Polish Collection—And This Is Our #1 Fave Color’ (*Hello Giggles*, 3 August 2016) <https://hellogiggles.com/lifestyle/home-decorating/kerrywashington-feminist-nail-polish/> accessed 20 August 2021.

47 Jasanoff (n 17).

48 S Wachter-Boettcher, *Technically Wrong: Sexist Apps, Biased Algorithms, and Other Threats of Toxic Tech* (Norton 2017) 20.

49 See Chatterjee (n 41).

50 N Stoljar, ‘Feminist Perspectives on Autonomy’ (*Stanford Encyclopaedia of Philosophy*, 11 December 2018) <https://plato.stanford.edu/entries/feminism-autonomy> accessed 20 August 2021.

51 Corbin (n 16) 338.

52 Lupton ‘Mastering Your Fertility’ (n 34) 87.

53 ibid 82.

54 S Sheldon, ‘The Decriminalisation of Abortion: An Argument for Modernisation’ (2016) 36 Oxf J Leg Stud 334, 343.

55 Jasanoff (n 17).

56 See Corbin (n 16).

57 E Brown, ‘The Femtech Paradox: How Workplace Monitoring Threatens Women’s Equity’ (2021) 61 Jurimetrics J 289.

58 See Mehrnezhad and Almeida (n 15).

59 The presence of bias in the design of algorithms is well documented, see Wachter-Boettcher (n 48).

60 See K Levy, ‘Intimate Surveillance' (2014) 51 Idaho Law Rev 679.

61 M Duane and others, ‘The Performance of Fertility Awareness-Based Method Apps Marketed to Avoid Pregnancy’ (2016) 29 J Am Board Fam Med 508; A Freis and others, ‘Plausibility of Menstrual Cycle Apps Claiming to Support Conception’ (2018) 6 Front Public Health 98.

62 S Johnson, L Marriott and M Zinaman, ‘Can Apps and Calendar Methods Predict Ovulation with Accuracy?’ (2018) 34 Cur Med Res Opin 1587.

63 S Earle and others, ‘Use of Menstruation and Fertility App Trackers: A Scoping Review of the Evidence’ (2020) 47 BMJ Sex Reprod Health 90.

64 M McHugh, ‘Does Femtech Give Users Control of Their Health or Take It Away?’ (*Ringer:Tech*, 18 March 2019) <http://www.theringer.com/tech/2019/3/18/18267094/femtech-female-health-appsmenstruation-fertility-trackers-clue-glow-ava> accessed 20 August 2021.

65 See Wachter-Boettcher (n 48) 37–40.

66 ibid 31.

67 See Mehrnezhad and Almeida (n 15).

68 See J Butler, *Gender Trouble* (Routledge 2002); Crenshaw (n 23).

69 Eg J Bell, ‘What It’s Like to Get Your Period When You’re Trans’ (*Clue*, 18 February 2019) <https://helloclue.com/articles/cycle-a-z/what-it's-like-to-get-your-period-when-you're-trans> accessed 20 August 2021.

70 Mehrnezhad and Almeida (n 15) 13.

71 ibid.

72 P Bjørn and M Menendez-Blanco, ‘FemTech: Broadening Participation to Digital Technology Development’ (2019) MM'19: Proc 27th ACM Int Conf Multimed 510.

73 Wachter-Boettcher (n 48).

74 Notably, individual groups discussed in intersectional literature deserve further depth than can be afforded here (eg the unique impacts femtech can have on different racial, socio-economic, and other groups) due to the regulatory focus of this article.

75 See Butler (n 68).

76 Corbin (n 16) 352.

77 A Weber and others, ‘Gender Norms and Health: Insights from Global Survey Data’ (2019) 393 The Lancet 32455.

78 S Taylor-Alexander and others, ‘Beyond Regulatory Compression: Confronting the Liminal Spaces of Health Research Regulation’ (2016) 8 Law Innov Technol 149.

79 Post Brexit the ICO website mentions the ‘UK GDPR’; however, at the time of writing this retains all same rights and principles but has implications for transfers between UK and EEA.

80 Regulation (EU) 2016/679, art 9.

81 ibid art 4(15).

82 Mehrnezhad and Almeida (n 15) 7.

83 See L Marelli, E Lievevrouw and I Van Hoyweghen, ‘Fit for Purpose? The GDPR and the Governance of European Digital Health’ (2020) 41 Policy Stud 447.

84 Mehrnezhad and Almeida (n 15) 12.

85 See Marelli and others (n 83).

86 Mehrnezhad and Almeida (n 15) 12.

87 ibid.

88 ibid 2.

89 By looking at privacy notices and tracking practices, see Mehrnezhad and Almeida (n 15).

90 Smartphone companies such as Apple have introduced notice and consent model for tracking across apps.

91 Mehrnezhad and Almeida (n 15) 2.

92 ibid 11.

93 ibid.

94 Medical Devices Regulations 2002 (SI 2002 No 618, as amended), reg 2(1).

95 See L Downey and M Quigley, ‘Software as a Medical Device: A Bad Regulatory Fit?’ (Everyday Cyborgs 2.0, 15 March 2021) <https://blog.bham.ac.uk/everydaycyborgs/2021/03/15/software-as-a-medical-device-a-bad-regulatory-fit/> accessed 20 August 2021.

96 MHRA, ‘Guidance: Medical Device Stand-Alone Software Including Apps (Including IVDMDs)’ (v1.08) (MHRA, updated 8 July 2021) <https://assets.publishing.service.gov.uk/government/uploads/system/uploads/attachment_data/file/999908/Software_flow_chart_Ed_1-08b-IVD.pdf> 25.

97 The 2002 Regulations implement EU dirs 93/42 on general medical devices, 90/385 on active implantable medical devices, and 98/79 on *in vitro* diagnostic medical devices.

98 See Medical Devices (Amendment, etc) (EU Exit) Regulations 2020.

99 MHRA ‘Guidance’ (n 96).

100 See Downey and Quigley (n 95).

101 Medical Devices Regulations 2002 (n 94).

102 See Downey and Quigley (n 95).

103 MHRA ‘Guidance’ (n 96).

104 ibid.

105 ibid. The guidance points to the European Commission’s ‘Borderline Devices Manual’ but this was withdrawn on 6 January 2021. Similar guidance is now available on the MHRA website.

106 A Eckersley, ‘Why We Are Removing the Fertile Window’ (*Clue*, 23 February 2021) <https://helloclue.com/articles/how-to-use-clue/why-we-are-removing-the-fertile-window> accessed 20 August 2021.

107 See Nuffield Council on Bioethics, *Novel Neurotechnologies: Intervening in the Brain* (NCB 2013) xxi.

108 Taylor-Alexander and others (n 78).

109 MHRA ‘Guidance’ (n 96).

110 Shemtob and Littlewood (n 11).

111 See Earle and others (n 63).

112 ASA, ‘ASA Ruling on NaturalCycles Nordic AB Sweden t/a Natural Cycles’ (*A*29 August 2018) <https://www.asa.org.uk/rulings/naturalcycles-nordic-ab-sweden-a17-393896.html> accessed 20 August 2021.

113 See Corbin (n 16).

114 See G Laurie, ‘Introduction: Towards a Learning Health Research Regulation System’ in G Laurie and others (eds), *The Cambridge Handbook of Health Research Regulation* (CUP 2021) 4–6; Academy of Medical Sciences, ‘A New Pathway for the Regulation and Governance of Health Research’ (AMS, 2011).

